# 787 A 13-year Experience with a 3-stage Dermal Regeneration Matrix Approach to Acute Hand Burns

**DOI:** 10.1093/jbcr/irae036.328

**Published:** 2024-04-17

**Authors:** Jose Antonio Arellano, Tiffany Jeong, Mario Alessandri-Bonetti, Hilary Liu, Sumaarg Pandya, Guy M Stofman, Francesco Egro

**Affiliations:** University of Pittsburgh Medical Center, Pittsburgh, PA; University of Pittsburgh Medical Center, Pittsburgh, PA; University of Pittsburgh Medical Center, Pittsburgh, PA; University of Pittsburgh Medical Center, Pittsburgh, PA; University of Pittsburgh Medical Center, Pittsburgh, PA; University of Pittsburgh Medical Center, Pittsburgh, PA; University of Pittsburgh Medical Center, Pittsburgh, PA

## Abstract

**Introduction:**

Dermal regeneration matrix (DRM) has been demonstrated to be safe and beneficial in improving functional outcomes for the management of acute hand burns. DRM followed by split thickness skin graft (STSG) allows for a two-stage reconstruction for most operative hand burn injuries. Our site routinely implements a 3-staged approach with cadaver allograft at the first stage. The aim of this study is to compare the surgical and functional outcomes of 2-staged DRM reconstruction and 3-staged reconstruction.

**Methods:**

A retrospective study was conducted to review surgical and functional outcomes of patients treated for hand burns. All patients seen from April 2009 and December 2022 with hand burns, for whom objective hand measurements were available, were considered for the study.

**Results:**

From 2009 to 2023, 227 patients were treated for hand burns. Of them, 44 patients had objective hand measurements and were included in the study. Patient (77.3% males,22.7% females) mean age was 39.3±16.1 and the mean BMI was 27.1±6.4. Patients suffered from 1.3±1.4 comorbidities. Patients were followed for 22.0±25.0 months. Most burns were full thickness (97.7%, n=43) and involved 15.3±16.7% TBSA. Patients suffered thermal injuries (93.2%, n=41) and electrical burns (6.8%, n=3). Many patients had bilateral hand involvement (52.3%, n=23).

Most cases (n=27) received a 3-staged approach with cadaver allograft at first stage, dermal regeneration matrix (DRM) at second stage, and split thickness skin graft (STSG) for final reconstruction. Fewer cases received a 2-staged approach with DRM and STSG (n=10). No 3-staged cases required repeat STSG, while 40% of 2-staged cases required repeat STSG. Repeat STSG in acute management was significantly associated with patients who received the 2-staged (p < 0.01).

There was no significant difference in mean baseline DASH scores for patients who received cadaveric allograft during acute management (46.5±22.3%) when compared to those who did not (48.7±22.5%) (p=0.8033).

**Conclusions:**

These data suggest a 3-stage approach may conserve autologous STSG, with fewer instances of repeat grafting. This approach may be especially useful when autologous skin is limited. Patients who received a 3-staged approach had comparable mean DASH scores when compared to patients who received a 2-staged approach, suggesting that a 3-staged approach does not diminish the functional benefits of DRM previously demonstrated in the literature.

**Applicability of Research to Practice:**

When autologous skin is limited for grafting complex burn patients, surgeons may consider a 3-stage approach to using DRM.

